# Supplementary figures and images for: Population Structure and Genetic Diversity of Cucurbita moschata Based on Genome-Wide High-Quality SNPs
Source: Plants (Basel). 2020 Dec 29;10(1):56. doi: 10.3390/plants10010056 (PMC7823833; doi:10.3390/plants10010056)

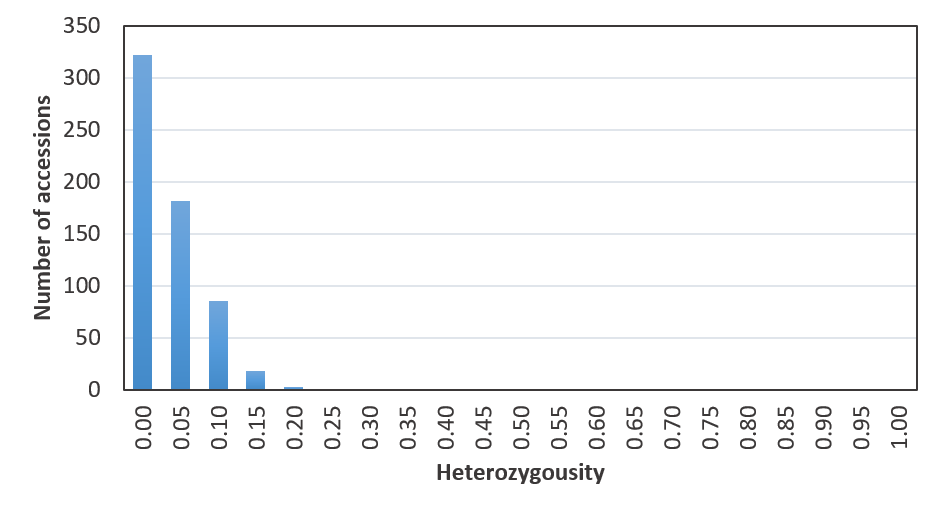

Supplement: Supplementary file 1 [file plants-10-00056-s001.zip › Fig S1.tif]

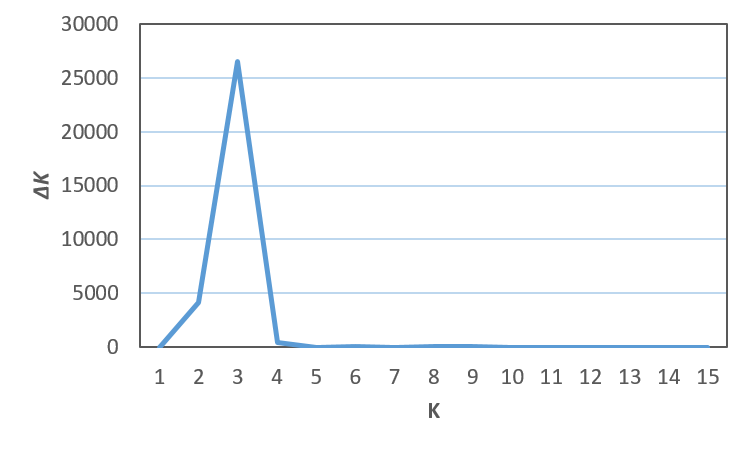

Supplement: Supplementary file 1 [file plants-10-00056-s001.zip › Fig S2.tif]

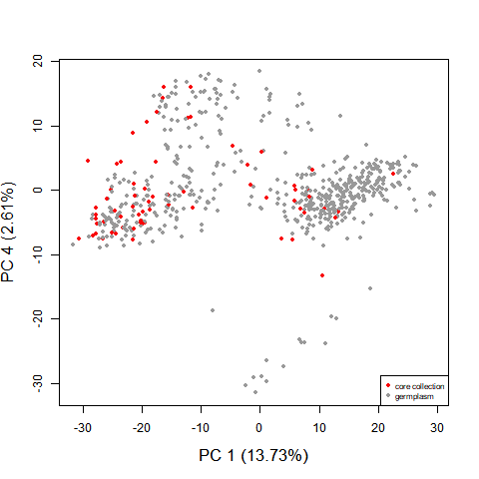

Supplement: Supplementary file 1 [file plants-10-00056-s001.zip › Fig S3.tif]
